# Will You Forgive Your Supervisor’s Wrongdoings? The Moral Licensing Effect of Ethical Leader Behaviors

**DOI:** 10.3389/fpsyg.2019.00484

**Published:** 2019-03-05

**Authors:** Rong Wang, Darius K.-S. Chan

**Affiliations:** ^1^College of Management, Shenzhen University, Shenzhen, China; ^2^Beijing Key Lab of Applied Experimental Psychology, Faculty of Psychology, Beijing Normal University, Beijing, China; ^3^Department of Psychology, The Chinese University of Hong Kong, Shatin, Hong Kong

**Keywords:** abusive supervision, ethical leadership, moral transgression, moral licensing, motivation

## Abstract

Moral licensing theory suggests that observers may liberate actors to behave in morally questionable ways due to the actors’ history of moral behaviors. Drawing on this view, a scenario experiment with a 2 (high vs. low ethical) × 2 (internal vs. external motivation) between-subject design (*N* = 455) was conducted in the current study. We examined whether prior ethical leader behaviors cause subordinates to license subsequent abusive supervision, as well as the moderating role of behavior motivation on such effects. The results showed that when supervisors demonstrated prior ethical behaviors, subordinates, as victims, liberated them to act in abusive ways. Specifically, subordinates showed high levels of tolerance and low levels of condemnation toward abusive supervision and seldom experienced emotional responses to supervisors’ abusive behaviors. Moreover, subordinates tended to attribute abusive supervision, viewed as a kind of mistreatment without an immediate intent to cause harm, to characteristics of the victims and of the organization rather than of the supervisors *per se*. When supervisors behaved morally out of internal rather than external motivations, the aforementioned licensing effects were stronger.

## Introduction

According to the 2013 National Business Ethics Survey in the United States, up to 60% of reported misconduct involved individuals with managerial authority, from supervisors to top management (Ethics Resource Center, 2013). The increasing prevalence of workplace misconduct and business scandals has driven public attention to the concept of ethical leadership, which refers to “the demonstration of normatively appropriate conduct through personal actions and interpersonal relationships, and the promotion of such conduct to followers through two-way communication, reinforcement, and decision-making” (Brown et al., 2005, p. 120). Previous research has shown that ethical leadership is beneficial to organizations. At the individual level, followers’ job satisfaction, job-related enthusiasm, and organizational commitment have been found to increase with ethical leadership (Avey et al., 2012), whereas intention to leave the workplace decreases (Kim and Brymer, 2011). Ethical leadership can also restrain negative workplace behaviors (Den Hartog and Belschak, 2012) and improve job performance and different forms of organizational citizenship behaviors (OCBs; Kalshoven and Boon, 2012). At the organizational level, ethical leadership is related to the ethical climate of an organization (Neubert et al., 2009), long-term competitive performance (Kim and Brymer, 2011), social capital (Pastoriza et al., 2008), and customer relations (Zheng et al., 2011).

Despite the apparent benefits of ethical leadership discussed above, it is difficult if not impossible for a leader to always be ethical or positive. Leader behaviors are inconsistent and even paradoxical in increasingly competitive organizational environments (Zhang et al., 2015). For example, Johnson et al. (2012) tracked the behaviors of 53 high-level managers over a period of 15 consecutive working days. They observed a negative but relatively weak correlation between leaders’ transformational and abusive behaviors (*r* = -0.25). More importantly, in their study, the proportion of within-person variance was 37% for transformational behavior, indicating leaders may not consistently maintain this transformational behavior. Lin et al. (2016) have further found that ethical leadership is likely to cause ego depletion, and displays of ethical leader behaviors are associated with an increase in abusive behavior the following day. Simply put, an ethical leader is likely to commit occasional moral transgressions that are questionable when judged by norms associated with workplace-related policies, procedures, or practices and/or with codes of interpersonal conduct (Shapiro et al., 2011, p. 412).

Moral licensing theory explains why leaders may engage in contrasting behaviors and why subordinates may tolerate or even accept leaders’ questionable actions to some extent. Moral licensing is defined as “people’s perception that they are permitted to take actions that could be seen as socially undesirable or morally questionable, due to history of moral behaviors” (Miller and Effron, 2010, p. 118). There are two potential mechanisms underlying the moral licensing effect: the moral credit model and the moral credential model. The moral credit model suggests that one’s behavioral history can offset or balance out future wrongdoings. Past moral behaviors increase actors’ moral credits, which make future bad deeds permissible even if these bad deeds are perceived as immoral. In addition to the moral licensing theory, some other theories and models including the idiosyncrasy credit theory (Hollander, 1958) and the moral balance model (Nisan, 1991) hold a similar view toward moral credit. Different from the moral credit model, which does not suggest that the perceived meaning of permitted bad deeds changes, the moral credential model indicates that individuals’ good behavioral histories provide a license for subsequent bad deeds by changing the way they are construed (Miller and Effron, 2010). In other words, according to the moral credential model, bad deeds are licensed because they are likely to be perceived as non-transgressions as a result of actors’ previous good deeds.

Existing research on moral licensing mainly concentrates on three topics of social psychology. (a) Discrimination or racism (e.g., Monin and Miller, 2001; Effron et al., 2009). For example, when non-sexist participants were presented an opportunity to build their moral credentials (e.g., disagreeing with sexist statements or selecting a member from the stereotyped group), they were more likely to commit sexist behaviors in subsequent tasks (Monin and Miller, 2001). (b) Prosocial behavior (Sachdeva et al., 2009; Jordan et al., 2011). Sachdeva et al. (2009) found that after writing a story referring to their own positive traits, individuals tended to donate less money and engage less in cooperative behaviors related to environmental protection. (c) Consumer decision (Khan and Dhar, 2006; Wilcox et al., 2009). For example, individuals were more likely to make indulgent food choices when a healthy item was also presented, especially those individuals with higher levels of self-control, because the presence of healthy food vicariously fulfills nutrition-related goals and provides a license to indulge (Wilcox et al., 2009).

Based on the findings and contributions of previous research, we expand the scope of moral licensing theory in the current study by applying it to the workplace, namely, leaders’ behaviors. In actuality, a relatively limited number of studies in the area of industrial and organizational psychology have been conducted under the framework of moral licensing (e.g., Castilla and Benard, 2010; Klotz and Bolino, 2012; Ormiston and Wong, 2013). Klotz and Bolino (2012) have proposed a model that suggests that employees’ prior OCB will license subsequent counterproductive workplace behavior (CWB), which will in turn inflict less harm on their personal reputation. Yam et al. (2017) provided further empirical evidence. They have found that employees who are compelled to engage in OCB hold a heightened sense of entitlement. Subsequently, however, these focal employees are likely to display deviant behaviors both within and outside of the organization. Moreover, it has been shown that companies’ previous corporate social responsibility (CSR) is positively related to their subsequent corporate social irresponsibility (CSiR), which is licensed because of the moral credits achieved through prior CSR (Ormiston and Wong, 2013). Castilla and Benard (2010) have also suggested that when an organizational culture promotes meritocracy, gender bias is pronounced because moral credentials are established by promoting meritocracy.

However, we take a subordinate-centric perspective instead of an actor-centric perspective in the present research. Specifically, we are interested in exploring whether leaders’ previous ethical behaviors cause subordinates to license subsequent morally questionable behaviors from the same leader. It is worth mentioning that the current research focuses on both moral and immoral behaviors displayed by a leader in the workplace. According to Effron and Monin (2010), when transgressions are blatant and when they occur in the same domain as the prior moral behaviors, they are likely to increase leaders’ levels of hypocrisy, making moral licensing unworkable. Thus, a leader’s moral transgressions are expected to be ambiguous. In this study, abusive supervision, which refers to “subordinates’ perceptions of the extent to which supervisors engage in sustained display of hostile verbal and non-verbal behaviors, excluding physical contact” (Tepper, 2000, p. 178), was selected as a representative example since abusive supervision does not include an immediate intent to cause harm (Tepper, 2007).

Various psychological perspectives were adopted to examine the hypothesized moral licensing effect in the current study. First, following Effron and Monin (2010), we directly assessed participants’ attitude to abusive supervision, namely, whether they would permit the ethical leader to engage in the subsequent moral transgression. Second, since moral emotions represent a key element of moral appraisals (Tangney et al., 2007), we also measured participants’ moral emotional responses to abusive behaviors of supervisors. Third, as mentioned earlier, abusive supervision is a kind of ambiguous moral transgressions without an immediate intent to cause harm (Tepper, 2007). We evaluated participants’ attribution styles related to abusive supervision to further explore the moral licensing effect.

### Permissibility

Moral licensing theory suggests that individuals perceive morally questionable and undesirable behaviors as acceptable or permitted if they have previously engaged in ethical behaviors (Miller and Effron, 2010). In addition to the actors *per se*, observers are likely to endow actors with moral credentials due to actors’ past history of ethical behaviors (Krumm and Corning, 2008). Thus, there are reasons to believe that leaders’ prior ethical behaviors, as a moral license, can increase subordinates’ levels of permissibility of subsequent abusive supervision. Based on previous studies (Effron and Monin, 2010), we chose subordinates’ tolerance and condemnation as two indexes of permissibility in our work. We expected a positive effect of leaders’ prior ethical behaviors on permissibility. The following hypothesis was posited.

Hypothesis 1a. Compared to situations when subordinates’ supervisors have not performed prior ethical behaviors, in situations when supervisors have performed prior ethical behaviors, subordinates will be more likely to tolerate subsequent abusive supervision, namely.

Hypothesis 1b. Compared to situations when subordinates’ supervisors have not performed prior ethical behaviors, in situations when supervisors have performed prior ethical behaviors, subordinates will be less likely to condemn abusive supervision and abusive supervisors.

### Moral Emotion

Leaders’ prior ethical behaviors also influence observers’ emotional responses to moral transgressions. Tangney et al. (2007) summarized two categories of moral emotions: self-conscious emotions and other-focused emotions. Self-conscious emotions (e.g., shame, guilt, embarrassment, and even pride) are evoked by self-reflection and self-evaluation when one is the *actor* of moral-related behaviors. Shortly, the self is the object of these self-conscious emotions. However, other-focused moral emotions, such as anger, contempt, disgust, elevation, and gratitude, are experienced when observing the admirable or undesirable deeds of others. Compared with the absence of prior ethical behaviors, the presence of ethical behaviors causes leaders’ transgressions to be more acceptable and, in turn, decreases the experience of other-focused moral emotions among observers. Therefore, leaders’ prior ethical behaviors should have a negative effect on subordinates’ moral emotional responses to abusive supervision. The following hypothesis was posited.

Hypothesis 1c. Compared to situations when subordinates’ supervisors have not performed prior ethical behaviors, in situations when supervisors have performed prior ethical behaviors, subordinates will be less likely to experience moral emotional responses to abusive supervision.

### Attribution

According to Effron and Monin (2010) and Miller and Effron (2010), when licensing and licensed behaviors belong to the same domain (e.g., workplace), and licensed behaviors are ambiguous, the moral credential model should be applied. That is to say, a good behavioral history licenses subsequent bad deeds by changing the way they are construed. Abusive supervision obscures perpetrators’ objectives and intentions (Tepper, 2000, 2007). This is why research has increasingly taken employees’ attributions into account when exploring the perception and consequences of abusive supervision (e.g., Martinko et al., 2011; Liu et al., 2012). When facing supervisors’ ambiguous mistreatment such as abusive supervision, the focal employees are likely to perceive them in different ways, as either positive or negative. They can attribute abusive supervision to both internal (e.g., personal disposition) and external (e.g., organizational influence) sources (Breaux et al., 2010) or to two distinct causal motives including performance promotion motives and injury initiation motives (Liu et al., 2012). It is expected that after building moral credentials because of leaders’ past ethical behaviors, employees will tend to construe abusive supervision in positive ways, namely, by attributing them to external factors such as characteristics of the victims and the organization rather than to the actors themselves. The following hypothesis was posited.

Hypothesis 1d. Compared to situations when subordinates’ supervisors have not performed prior ethical behaviors, in situations when supervisors have performed prior ethical behaviors, subordinates will exhibit a greater tendency to attribute subsequent abusive supervision in positive ways. Specifically, they will be more likely to attribute abusive supervision to characteristics of the victims and the organization, and less likely to attribute it to the supervisors *per se*.

Admittedly, theories such as the cognitive dissonance theory (Festinger, 1957) support behavioral consistency rather than compensation. The moral licensing theory has also maintained that individuals tend to keep their behaviors consistent under certain conditions: namely, when actors hold high levels of moral identity, when prior behaviors demonstrate commitment to the goal rather than just the progress to the goal, and/or when actors are inclined to avoid the potential hypocrisy caused by inconsistent behaviors (Miller and Effron, 2010). Therefore, research has recently begun to explore influential factors that distinguish behavioral consistency and compensation. For example, it has been found that the choice of moral compensation or moral consistency depends on the reactive or a proactive perspective taken by the actor. Cognitive depletion that results in a reactive approach makes moral compensation preferable, whereas moral consistency prevails when cognitive resources are available that lead to a proactive approach (Joosten et al., 2013). Moral licensing research related to consumer choice has also indicated that a prior choice that has activated a positive self-concept can license a more self-indulgent choice among available options. However, the preference for such indulgence diminishes when the licensing task is driven by external motivations (Khan and Dhar, 2006).

Inspired by existing findings, the present research also aimed to explore the boundary conditions of moral licensing effect by taking behavioral motivation into account. Behavioral demonstrations are likely to be motivated by different factors such as internal virtues or external pressures. Thus, researchers have debated a great deal whether behavior-based or virtue-based instruments should be employed to assess ethical leadership (Brown et al., 2005; Riggio et al., 2010). Indeed, the motivation issue has been put forth in other positive topics of leadership, such as pseudotransformational leadership and personalized charismatic leadership (Howell and Avolio, 1992; Bass and Steidlmeier, 1999). In other words, leaders’ ethical behaviors are not always perceived to be positive.

Since ethical leadership is a value-driven form of leadership (Brown et al., 2005), behavioral motivation is as important as behavioral demonstrations. The perceived motivations behind supervisors’ prior moral behaviors will influence the licensing effects. Existing research has revealed that the relationship between ethical leadership and employees’ work engagement is weaker when leaders are perceived as Machiavellians, who tend to seek opportunities for impression management and personal benefits (Becker and Dan O’ Hair et al., 2007; Den Hartog and Belschak, 2012). Following the same logic, when leaders’ prior ethical behaviors are perceived as compulsory because of external forces such as organizational policies, employees should be reluctant to endorse leaders with moral credentials. The hypothesized licensing effects will be weakened accordingly. Thus, we further hypothesize the following.

Hypothesis 2. When subordinates perceive that prior ethical behaviors are driven by internal motivation (vs. external motivation), the moral licensing effects posited in our first set of hypotheses will be stronger (vs. weaker).

## Materials and Methods

### Participants

We recruited participants from evening classes designed for full-time workers in Beijing, China. A total of 455 valid data was collected. Sixty-nine percent of the participants were female (*N* = 313). Their average age was 27.20 years (*SD* = 5.05), and the average tenure was 5.63 years (*SD* = 4.56). Most of the participants (*N* = 397) had a bachelor’s degree or higher, and they worked in various occupations, e.g., HR, teacher, and sales.

### Procedure

We used a 2 × 2 between-subject design to manipulate a supervisor’s prior behaviors as well as behavioral motivation. Participants were randomly assigned to one of four conditions: high ethical-internal motivation (*N* = 148), high ethical-external motivation (*N* = 102), low ethical-internal motivation (*N* = 99), and low ethical-external motivation (*N* = 106).

They first read a story describing a supervisor’s behaviors (high or low ethical) with different behavioral motivations (internal or external motivation). The vignette was adapted from Bhal and Dadhich (2011) (details can be seen from Appendix). Following the story, they indicated the extent to which the supervisor’s behaviors were ethical (1 = “totally unethical”; 5 = “totally ethical”), and the extent of their agreement that such behaviors were driven by internal motivation (1 = “nothing to do with the internal motivation” to 5 = “due to the internal motivation completely”) and external motivation (1 = “nothing to do with the external motivation” to 5 = “due to the external motivation completely”). These questions were used for manipulation checks.

Then, the description of abusive supervision (Wang and Jiang, 2015) was shown to participants, followed by several questions that addressed their (a) tolerance, (b) condemnation, (c) moral emotional responses, and (d) attributions to such abusive behaviors. They were reminded that all the questions should be answered from the perspective of a subordinate in the story. Finally, we collected the participants’ demographic information and debriefed the purpose of the study after they completed the experiment. A small incentive (of a value of approximately $1) was given as a token of appreciation.

### Measures of Dependent Variables

#### Tolerance

One question was used to measure the participants’ tolerance of the abusive supervision: “Based on a five-point scale (1 = ‘totally intolerable’; 5 = ‘totally tolerable’), please indicate to what extent you can tolerate what the supervisor is doing.”

#### Condemnation

Nine items adapted from Effron and Monin (2010) were used to measure the participants’ levels of condemnation of the abusive supervision (four items, such as “The behavior was wrong”) as well as to the actor (five items, such as “The supervisor in the story should be blamed”). Participants answered these questions based on a five-point scale anchored from 1 = “strongly disagree” to 5 = “strongly disagree.” Cronbach’s α coefficients were 0.71 (condemnation to the behavior) and 0.85 (condemnation to the actor).

#### Moral Emotion

Based on a five-point scale (1 = “totally impossible”; 5 = “totally possible”), participants indicated to what extent they would experience the five kinds of other-focused moral emotions (i.e., anger, contempt, disgust, elevation, and gratitude; Tangney et al., 2007), if the supervisor were to engage in such actions. The two positive emotions, namely, elevation and gratitude, were coded reversely. Cronbach’s α coefficient was 0.77.

#### Attribution

First, seven items from Burton et al. (2014) were used to assess self-attribution (four items, such as “The source of the supervisor’s behavior reflects something about me”) and supervisor-attribution (three items, such as “The cause of the supervisor’s behavior is something controllable by the supervisor”). Then, three items cited from Breaux et al. (2010) were used to assess organization-attribution, such as “The supervisor works under a lot of pressure from the organization.” All the items were rated on a five-point scale ranging from 1 = “strongly disagree” to 5 = “strongly agree.” Cronbach’s α coefficients were 0.74 (self-attribution), 0.60 (supervisor-attribution), and 0.59 (organization-attribution).

## Results

### Pilot

To ensure that the abusive scenario was ambiguous, we distributed the experimental materials to 109 full-time employees prior to data collection. Sixty-eight percent were female (*N* = 74). Their average age was 29.34 years (*SD* = 5.77), and the average length of employment was 5.82 years (*SD* = 5.62). The definition of abusive supervision was stated at the beginning of the materials. Then, participants indicated to what extent the described situation was abusive and hostile based on a 10-point Likert scale (1 = “not at all,” 10 = “yes, completely”). Moreover, to explore the potential attribution styles of the designed scenario, we required participants to write down all of the possible explanations for the situation, by asking, “In addition to the abusive supervision, do you think there is any other explanation for the supervisor’s behaviors in the story? Please write them down.”

The results of the descriptive analysis showed that the rating was approximately five (*M* = 4.97, *SD* = 2.41), indicating the ambiguity of the abusive scenario. Moreover, after coding the explanations provided by the participants, self-, organization-, and supervisor-attribution were found to be the three main attribution styles for abusive supervision. These findings were consistent with prior studies related to the attribution styles of abusive supervision (Martinko et al., 2011; Burton et al., 2014).

### Manipulation Checks

First, the results of an independent sample *t*-test showed that participants under the high ethical condition (*M* = 3.76, *SD* = 0.93) considered the supervisor in the story to be more ethical than those under the low ethical condition (*M* = 2.42, *SD* = 1.08), *t* = -14.04, *df* = 403, *p* < 0.001. Then, based on two questions about motivation, multivariate analysis of variance (MANOVA) was conducted, and the results indicated a significant main effect of motivation, *F*(2,451) = 11.15, *p* < 0.001. Specifically, compared with those under the external motivation condition, participants under the internal motivation condition reported higher levels of internal motivation, *F*(1,452) = 7.29, *p* < 0.01, and lower levels of external motivation, *F*(1,452) = 17.99, *p* < 0.001. Therefore, as expected, the manipulations of behavior and motivation were successful.

### Hypotheses Test

Our data are completely derived from self-reports, and common method bias (CMB) (Podsakoff et al., 2003) is a potential problem. We conducted a confirmatory factor analysis (CFA) to evaluate the goodness of fit for the seven-factor model prior to examining the hypotheses (the manipulated variables were not included). Results revealed satisfactory psychometric properties of the measurement model: χ^2^ = 642.27, *df* = 249, IFI = 0.91, CFI = 0.91, RMSEA = 0.06 (90% CI: 0.05, 0.07), indicating that the survey items accurately captured each of the constructs examined.

Table 1 presents the results of descriptive statistics and zero-order correlations of variables. We found that educational levels were significantly correlated to participants’ attribution styles, indicating that it was a potential covariate. Therefore, multivariate analysis of covariance (MANCOVA) was first used to test the main effect of the supervisor’s ethical behavior and the interaction effect of ethical behavior × behavioral motivation on our outcomes (i.e., tolerance, condemnation, moral emotion, and attribution), by treating educational levels as a control variable. The results revealed that both the main effect of ethical behavior, *F*(7,434) = 48.83, *p* < 0.001, and the interaction effect of ethical behavior × behavior motivation, *F*(7,434) = 2.82, *p* < 0.01, were significant. Then, we conducted a series of 2 × 2 analysis of variance (ANOVA) for tolerance, condemnation, and moral emotions. Also, a series of 2 × 2 analysis of covariance (ANCOVA) were employed for three types of attribution.

**Table 1:** Descriptive statistics and zero-order correlations of variables.

Variable	*M*	*SD*	1	2	3	4	5	6	7	8	9	10
1 Age	27.20	5.05										
2 Gender	0.70	0.46	-0.10									
3 Tenure (year)	5.63	4.56	0.83^**^	-0.10^*^								
4 Education	2.03	0.53	0.25^**^	-0.06	0.01							
5 Tolerance	3.31	1.11	0.07	0.04	0.02	0.05						
6 Condemnation (actor)	3.05	0.99	-0.06	-0.05	-0.01	-0.03	-0.39^**^					
7 7 Condemnation (AS)	2.59	0.83	-0.08	-0.06	-0.06	-0.00	-0.50^**^	0.52^**^				
8 Moral emotion	3.50	0.86	-0.04	0.01	-0.01	0.05	-0.49^**^	0.53^**^	0.47^**^			
9 Self-attribution	3.49	0.80	0.03	-0.06	0.01	0.10^*^	0.33^**^	-0.30^**^	-0.31^**^	-0.23^**^		
10 Organization-attribution	2.86	0.86	0.05	-0.06	-0.04	0.17^**^	0.16^**^	-0.23^**^	-0.09^*^	-0.15^**^	0.21^**^	
11 Supervisor-attribution	3.33	0.79	0.04	-0.05	0.04	0.13^**^	-0.16^**^	0.17^**^	0.22^**^	0.40^**^	0.08	0.19^**^


*Note. AS = abusive supervision; gender (treated as a dummy variable): 0 = male, 1 = female.*

*Education (treated as the ordinal data): 1 = high school and below, 2 = bachelor, 3 = master, 4 = doctor. *N* = 455. ^∗^*p* < 0.05; ^∗∗^*p* < 0.01.*

#### Tolerance

**FIGURE 1:**
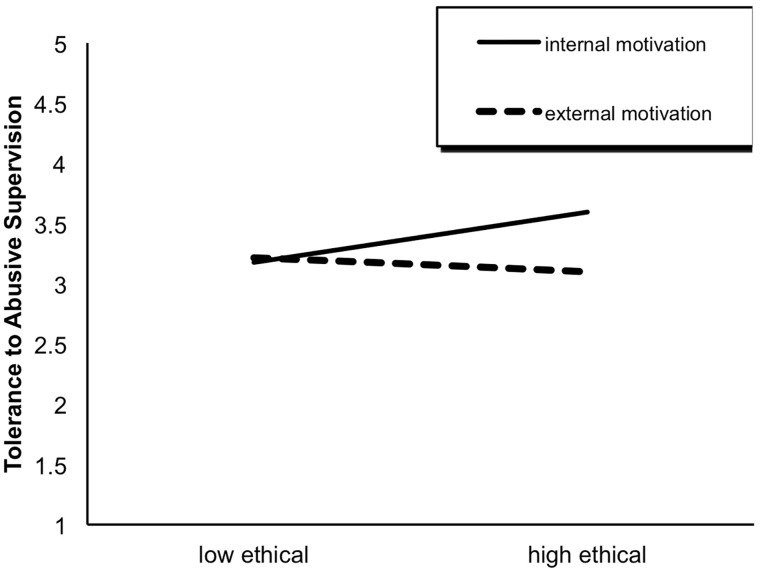
The interaction effect of ethical behavior × behavioral motivation on tolerance to abusive supervision.

The results of the ANOVA yielded a significant interaction effect of ethical behavior × behavioral motivation on tolerance, *F*(1,444) = 6.53, *p* < 0.05. As shown in Figure 1, when the supervisor had engaged in previous ethical behaviors, subordinates were more likely to tolerate the subsequent abusive supervision, but this difference was significant only for the internal motivation condition (internal motivation: *t* = -3.00, *df* = 242, *p* < 0.01; external motivation: *t* = 0.77, *df* = 202, *p* > 0.05).

#### Condemnation

**FIGURE 2:**
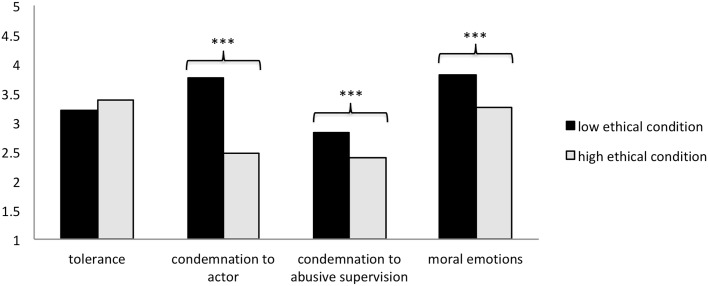
The influences of prior ethical behavior on subsequent abusive supervision. ^∗∗∗^*p* < 0.001.

**FIGURE 3:**
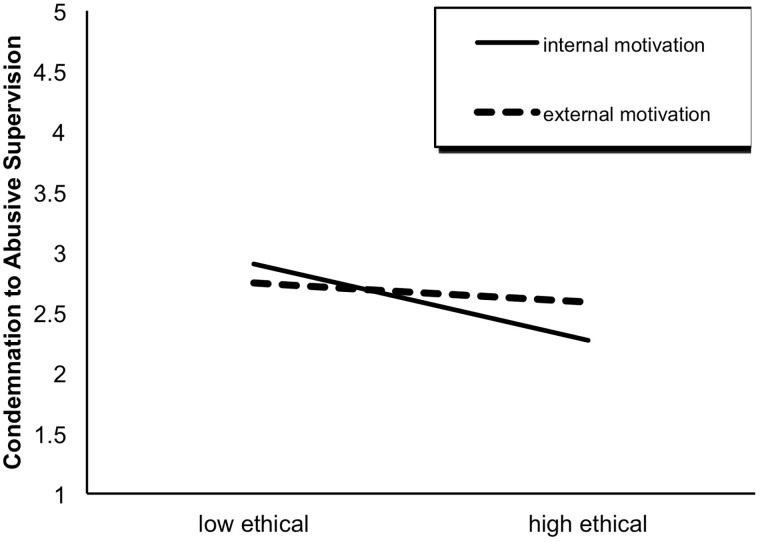
The interaction effect of ethical behavior × behavioral motivation on condemnation to abusive supervision.

We observed that both the main effect of ethical behavior, *F*(1,451) = 26.95, *p* < 0.001, and the interaction effect of ethical behavior × behavioral motivation, *F*(1,451) = 10.02, *p* < 0.01, were significant. Specifically, participants in the high ethical group (*M* = 2.40, *SD* = 0.74) were less likely to condemn the subsequent abusive supervision than their counterparts in the low ethical group (*M* = 2.82, *SD* = 0.88; see Figure 2). Furthermore, this difference was only significant for the internal motivation condition (internal motivation: *t* = 5.58, *df* = 180, *p* < 0.001; external motivation: *t* = 1.48, *df* = 206, *p* > 0.05; see Figure 3).

**FIGURE 4:**
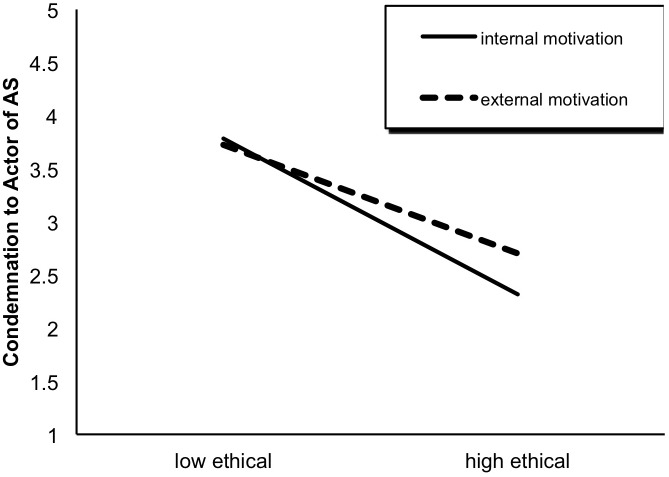
The interaction effect of ethical behavior × behavioral motivation on condemnation to the actor.

Following a similar pattern, a 2 × 2 ANOVA of participants’ condemnation of the actor yielded a significant main effect of ethical behavior, *F*(1,451) = 303.67, *p* < 0.001, as well as a significant interaction effect of ethical behavior × behavioral motivation, *F*(1,451) = 9.78, *p* < 0.01. Specifically, the participants in the high ethical group (*M* = 2.48, *SD* = 0.80) were less likely to condemn the abusive supervisor than their counterparts in the low ethical group (*M* = 3.75, *SD* = 0.71; see Figure 2). This difference was significant for both the internal (*t* = 14.62, *df* = 245, *p* < 0.001) and external motivation conditions (*t* = 10.15, *df* = 206, *p* < 0.001). However, the effect of ethical behavior on condemnation of the actor was stronger among the participants in the internal motivation group (vs. the external motivation group; see Figure 4).

#### Moral Emotion

The results showed a significant main effect of ethical behavior, *F*(1,451) = 46.06, *p* < 0.001. The participants in the high ethical group (*M* = 3.26, *SD* = 0.79) were less likely to experience moral emotions caused by abusive supervision than their counterparts in the low ethical group (*M* = 3.81, *SD* = 0.84; see Figure 2). We did not observe a significant interaction effect of ethical behavior × behavioral motivation on participants’ moral emotion, *F*(1,451) = 1.84, *ns*.

#### Attribution

**FIGURE 5:**
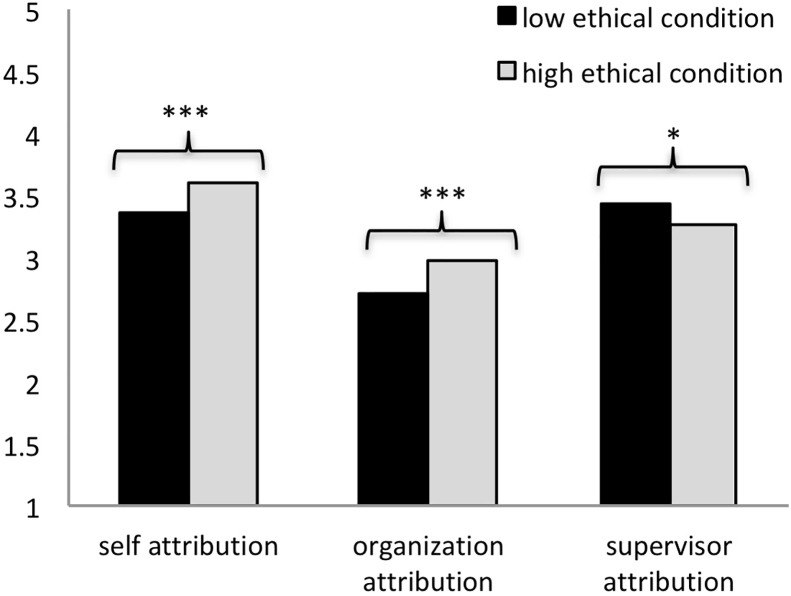
The influences of prior ethical behavior on attribution styles. ^∗^*p* < 0.05; ^∗∗∗^*p* < 0.001.

**FIGURE 6:**
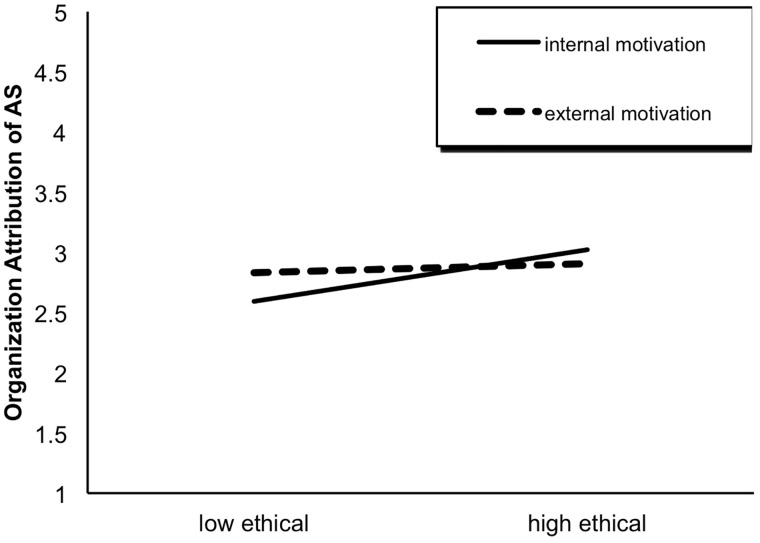
The interaction effect of ethical behavior × behavioral motivation on organizational attribution.

After controlling participants’ educational levels, the results of ANCOVA yielded a significant main effect of ethical behavior on all three types of attribution: self-attribution, *F*(1,440) = 10.68, *p* < 0.01; supervisor-attribution, *F*(1,440) = 4.60, *p* < 0.05; organization-attribution, *F*(1,440) = 7.66, *p* < 0.01. Specifically, compared with those in the low ethical condition, participants in the high ethical condition were more likely to attribute abusive supervision to themselves, i.e., victims (high ethical: *M* = 3.60, *SD* = 0.73; low ethical: *M* = 3.35, *SD* = 0.87) and organizations (high ethical: *M* = 2.97, *SD* = 0.81; low ethical: *M* = 2.72, *SD* = 0.89), and less likely to attribute it to the supervisor *per se* (high ethical: *M* = 3.26, *SD* = 0.76; low ethical: *M* = 3.42, *SD* = 0.81; see Figure 5). Moreover, for organization-attribution, the interaction effect of ethical behavior × behavioral motivation was significant, *F*(1,440) = 4.41, *p* < 0.05. As seen from Figure 6, the effect of ethical behavior on organization-attribution was significant only when leaders’ prior ethical behaviors were due to internal (*t* = -3.86, *df* = 187, *p* < 0.001) rather than external motivation (*t* = -0.62, *df* = 206, *p* > 0.05).

## Discussion

This research extends the topic of moral licensing to the area of leadership. Indeed, similar observer-licensing effects have also been demonstrated in previous studies. For example, Krumm and Corning (2008) found that heterosexual participants tended to rate ambiguously discriminatory behaviors as less discriminatory if the actors’ past actions were non-prejudicial (e.g., announcing one’s relationships with gay people). Our findings provide evidence that supports the existence of an observer-licensing effect in the workplace. If supervisors have demonstrated ethical behaviors in the past, especially when prior ethical behaviors were inspired by internal motivations, subordinates, even if they are victims, tend to liberate the same supervisors to behave in morally disreputable ways. Specifically, in our study, subordinates held high levels of tolerance and low levels of condemnation toward abusive supervision and seldom experienced emotional responses to supervisors’ abusive behaviors. Moreover, subordinates tended to attribute abusive supervision to characteristics of the victims and organization rather than to the supervisor *per se*.

Moral licensing theory maintains moral credits and moral credentials are potential underlying mechanisms of moral licensing effect. Two important variables determine which model will be applied: the ambiguity of the licensed behaviors and the domains of licensing and licensed behaviors. Effron and Monin (2010) have pointed out that good behavioral history cannot only license people to perform morally questionable behaviors in the same domain, but also in unrelated domains. In the current study, both supervisors’ prior ethical behaviors and subsequent moral transgression (i.e., abusive supervision) were in the workplace. If the previous moral behaviors and the subsequent licensed behaviors belong to the same domain (e.g., workplace), there are two possibilities. First, when the subsequent licensed behaviors are blatant transgressions, the moral licensing effect will not work and inconsistent behaviors will increase actors’ levels of hypocrisy. Second, when the subsequent licensed behaviors are ambiguous transgressions, only the moral credential model will work. That is, good behavioral history provides actors with a credential that serves to liberate their ambiguous moral transgressions by changing how the observers construe the transgression. Thus, we have designed abusive scenario as ambiguous and explored subordinates’ construal through evaluating their different attribution styles. Our findings support this assumption. If a leader demonstrates prior ethical behaviors, the subordinate will consider the leader’s subsequent ambiguous moral transgressions to be right, and find extra reasons (e.g., self-blame and organizational stress) to justify their “wrongdoings.” However, it is worth mentioning that performing ethical behaviors does not imply that leaders will definitely commit subsequent transgressions or that they can engage in transgressions with impunity, but only indicates that the subsequent ambiguous actions are likely to be interpreted as appropriate or moral.

Moreover, supervisors and subordinates belong to the same working teams. It is likely that subordinates license abusive supervision due to their shared membership with the supervisor. Social identity theory (Tajfel and Turner, 1979) suggests that people display positive bias to in-group members. They tend to judge in-group members more favorably than out-group members. Therefore, moral credential is endorsed more easily among in-group members (e.g., between a supervisor and a subordinate) than out-group members (e.g., strangers). That is why in Krumm and Corning’s (2008) study, the moral licensing effect found among heterosexual participants (i.e., in-group members) was attenuated among LGB (lesbian, gay, and bisexual) participants (i.e., out-group members).

We also found that when subordinates perceived that supervisors’ prior ethical behaviors were motivated by internal rather than external motivation, the moral licensing effects were stronger. As raters, subordinates’ cognition, together with supervisors’ behavior *per se*, may jointly contribute to subordinates’ subjective perceptions and decisions (Lord et al., 1980). For example, helping behaviors are not always rewarded. Several factors affect raters’ evaluation and reward decision, including ratees’ reputations (label), raters’ attribution of motives (cognitive factor) and liking (affective factor). When raters attribute ratees’ helping behaviors to altruistic motivations and have a higher level of liking for them, they are likely to distribute more rewards (Johnson et al., 2002). Therefore, compared with morality imposed by extrinsic factors, perceived intrinsic morality has more power to show that the supervisor is a good person. Based on this cognition, subordinates are inclined to endorse moral credentials and redefine the meaning of the action taken in the second situation (Monin and Miller, 2001).

### Managerial Implications

The main findings of this research have some implications for organizational practice. Subordinates have a tendency to liberate leaders’ morally questionable behaviors after observing leaders’ prior ethical behaviors, which may tolerate and even encourage the existence of destructive leadership styles. First, organizations can take steps including training and interventions to strengthen ethical climate. Organizations’ ethical climate is not only helpful to manage the ethical behaviors within the organizations, but also has impact on shaping organizational members’ zero-tolerance attitude to leaders’ mistreatments and questionable behaviors (Bartels et al., 1998). Second, organizations should provide supportive and fair organizational conditions to encourage employees’ internal whistle blowing [e.g., online whistle-blowing reporting systems (WBRS)] and to protect employees from retaliation for whistle blowing (Lowry et al., 2013). Third, subordinates’ right to appeal should be recognized once they come across the inappropriate actions from leaders. For example, human resource management department can build effective channels for complaint and appeal, and make the clear principle of appeal, in order to protect the lawful rights of employees and to assure the timely communication between leaders and subordinates.

Leaders should also pay continuous attention on forming and developing their own ethical values, and even modify personal values according to business ethics when it is necessary. Moreover, organizations can take roles in improving leader ethics. On the one hand, they should recognize the importance of ethical actions as well as ethical leaders, and encourage managerial authorities to add a virtue perspective as a complement to act-oriented perspectives when evaluating their ethical behaviors (Whetstone, 2001). On the other hand, they should consider ethics programs as part of manager’s management system, to emphasize the importance of ethics and to deepen understanding about ethics among leaders (Brenner, 1992).

### Limitations and Future Directions

The current research has several limitations that are worth mentioning. The first one concerns CMB due to experimental design. Experimental scenarios are deemed to be appropriate for the studies related to negative and sensitive topics (e.g., abusive supervision and ethical leadership; Bhal and Dadhich, 2011). It has also been suggested that the scenario experiment approach is an alternative research strategy in which a large sample of subjects is not necessarily required (Ehrhart and Naumann, 2004). Meanwhile, there are some weaknesses by using vignettes, such as relatively low external validity as well as CMB from self-report data and potential social desirability. However, the observed research findings are not greatly influenced for the following reasons. First, we found that the survey items captured each of the constructs well by applying the CFA technique. Second, in terms of the response bias caused by potential social desirability. We tested the mean scores of the participants’ responses to the dependent variables and there was no ceiling effect identified, indicating that they did not show generosity in answering most of the questions. Furthermore, it is believed that if social desirability did exist, regardless of the absence or presence of prior ethical behaviors, participants would maintain their attitude against abusive supervision. Thus, the moral licensing effect and the moderating effect of motivation would not occur. In the future, a socially desirable response scale should be included in the experiment. The impact of social desirability can then be excluded by assessing the correlation between participants’ scores on the social desirability scale and their responses to ambiguously immoral vignettes (Krumm and Corning, 2008). More importantly, we plead for the future filed study to replicate our findings by gathering data from multiple different sources in order to avoid common method effects.

Moreover, tolerance, as one of indexes of permissibility, was assessed by one item in this study. Indeed, single item measurements have also been discussed in other topics of organizational psychology including job satisfaction and organizational identification (Shamir and Kark, 2004). It has been suggested that a single-item measure may suffice when the measured construct is sufficiently narrow or is unambiguous to the respondent. Practical limitations, namely, space of the questionnaire, also favor the use of a single-item measure (Wanous et al., 1997). However, it is hoped that a well-constructed scale can be employed in the future.

Finally, our work also outlines three more directions for future research. First, using moral licensing theory as the overarching framework, we focused on leaders’ potential inconsistent behaviors from a subordinate-centric perspective. We explored participants’ attributions about leaders’ ambiguous abusive behaviors in order to find evidence to support moral credential model. Future researcher can attempt to examine the underlying mechanisms of moral licensing effect by including moral credits and moral credential into research model directly (Lin et al., 2016). Second, this study simply explored whether subordinates liberated leaders to conduct abusive behaviors due to the existence of past good deeds. Observing actions of others can also influence the observer’s self-perception (Goldstein and Cialdini, 2007). That is, it is possible that subordinates license not only leaders’ moral transgressions but also their own moral transgressions after observing leaders’ ethical behavior. Such vicarious licensing effect also deserves future attention (Kouchaki, 2011). Third, the present research emphasized the internal balance of individuals’ moral behaviors. In addition to moral licensing, the moral cleansing effect states that behaving immorally first urges people to engage in subsequent moral behaviors to regain lost worth (Sachdeva et al., 2009; Zhong et al., 2009). Therefore, another line of research of moral cleansing is also encouraged in the future to clarify the existence and influence of leader behavioral compensation.

## Ethics Statement

All procedures performed in studies involving human participants were in accordance with the ethical standards of the institutional and/or national research committee and with the 1964 Helsinki declaration and its later amendments or comparable ethical standards. Prior to the research, ethical approval was obtained from the Academic Ethics Committee of the Faculty of Psychology at Beijing Normal University. All individual participants were required to read and sign the informed consent before participating in this research.

## Author Contributions

RW designed the study, collected and analyzed the data, and wrote and revised the manuscript. DC reviewed the manuscript critically.

## Conflict of Interest Statement

The authors declare that the research was conducted in the absence of any commercial or financial relationships that could be construed as a potential conflict of interest.

## Appendix: The Vignettes Used in This Study

Please imagine that you are a subordinate in a food-processing factory located in Beijing, China, and X is your supervisor. The following stories describe the behavioral style of X as well as the daily interactions between you and X. Please read the stories carefully and then answer all the questions from the perspective of the subordinate in the stories.

### High Ethical-Internal Motivation

X is a person who can be trusted and who makes fair and balanced decisions and conducts his/her personal life in an ethical manner. X defines success not only by the result but also by an emphasis on the process. X also takes others’ opinions into consideration before making any decisions. X listens to the employees and disciplines those who violate ethical standards. X sets an example of how to do things the right way in terms of ethics. X has the best interest of employees in mind and discusses business ethics and values with the employees.

X always acts in ethical manner since X believes ethics is at the heart of business, and it is important for a leader to demonstrate ethical behaviors and to focus employees’ attention on these desirable behaviors.

### High Ethical-External Motivation

X is a person who can be trusted. X makes fair and balanced decisions and conducts his or her personal life in an ethical manner. X defines success not only by the result but also by an emphasis on the process. X also takes others’ opinions into consideration before making any decisions. X listens to the employees and disciplines those who violate ethical standards. X sets an example of how to do things the right way in terms of ethics. X has the best interest of employees in mind and discusses business ethics and values with the employees.

X always acts in ethical manner since your company is launching an ethics and compliance program after the media exposed so many business scandals to the public. To restrain inappropriate leader behaviors, your company has linked performance evaluation of a leader to ethics, and each leader will be penalized if employees complain about their ethics.

### Low Ethical-Internal Motivation

X is a person who cannot be trusted. X does not make fair and balanced decisions and does not conduct his or her personal life in an ethical manner. X defines success only in terms of results without any concern for how the results are achieved. X also does not take others’ opinions into consideration before making any decisions. X does not listen to the employees and does not discipline those who violate ethical standards. X does not care about the employees and never talks about business ethics or values with the employees.

X always acts in this manner since X believes ethics has nothing to do with business, and it is not at all important for a leader to demonstrate ethical behaviors or to focus employees’ attention on these desirable behaviors.

### Low Ethical-External Motivation

X is a person who cannot be trusted. X does not make fair and balanced decisions and does not conduct his or her personal life in an ethical manner. X defines success only in terms of results without any concern for how the results are achieved. X also does not take others’ opinions into consideration before making any decision. X does not listen to the employees and does not discipline those who violate ethical standards. X does not care about the employees and never talks about business ethics or values with the employees.

X always acts in this manner since your company believes benefits or profits are at the heart of business. He or she always stresses this in front of each member of the company, especially those in managerial positions. Moreover, your company has linked leaders’ salaries to their performance or productivity of themselves and that of their team. When a great deal of attention has to be paid to benefits, issues related to ethics are inevitable ignored inevitably.

### Abusive Supervision

One day, there is something wrong with your computer before an important job interview. Unfortunately, all of the applicants’ resumes and related materials are stored in your computer. The arranged interview is beginning very soon, and every staff member is feeling worried and upset about this difficult problem. After seeing and understanding the situation, X walks over to you and shouts angrily “Is this your first day working here? Are you too stupid to copy these important things?! If you make the same mistake in the future, I will never forgive you!”

## References

[B1] Avey J. B. Wernsing T. S. Palanski M. E. (2012). Exploring the process of ethical leadership: the mediating role of employee voice and psychological ownership. *J. Bus. Ethics* 107 21–34. 10.1007/s10551-012-1298-2

[B2] Bartels K. K. Harrick E. Martell K. Strickland D. (1998). The relationship between ethical climate and ethical problems within human resource management. *J. Bus. Ethics* 17 799–804. 10.1023/A:1005817401688

[B3] Bass B. M. Steidlmeier P. (1999). Ethics, character, and authentic transformational leadership behavior. *Leadersh. Q.* 10 181–217. 10.1016/S1048-9843(99)00016-8

[B4] Becker J. A. H. Dan O’ Hair H. (2007). Machiavellians’ motives in organizational citizenship behavior. *J. Appl. Commun. Res.* 35 246–267. 10.1080/00909880701434232

[B5] Bhal K. T. Dadhich A. (2011). Impact of ethical leadership and leader–member exchange on whistle blowing: the moderating impact of the moral intensity of the issue. *J. Bus. Ethics* 103 485–496. 10.1007/s10551-011-0876-z

[B6] Breaux D. M. Tepper B. J. Carr J. C. Folger R. G. (2010). “An attributional analysis of employees’ responses to abusive supervision,” in *The Dark Side of Management*, eds Neider L. L. Schriesheim C. (Charlotte, NC: Information Age Publishing), 69–92.

[B7] Brenner S. N. (1992). Ethics programs and their dimensions. *J. Bus. Ethics* 11 391–399. 10.1007/BF00870551

[B8] Brown M. E. Treviño L. K. Harrison D. A. (2005). Ethical leadership: a social learning perspective for construct development and testing. *Organ. Behav. Hum. Decis. Process.* 97 117–134. 10.1016/j.obhdp.2005.03.002

[B9] Burton J. P. Taylor S. G. Barber L. K. (2014). Understanding internal, external, and relational attributions for abusive supervision. *J. Organ. Behav.* 35 871–891. 10.1002/job.1939

[B10] Castilla E. J. Benard S. (2010). The paradox of meritocracy in organizations. *Adm. Sci. Q.* 55 543–576. 10.1097/ACM.0000000000002388 30067539 PMC6309930

[B11] Den Hartog D. N. Belschak F. D. (2012). Work engagement and machiavellianism in the ethical leadership process. *J. Bus. Ethics* 107 35–47. 10.1007/s10551-012-1296-4

[B12] Effron D. A. Cameron J. S. Monin B. (2009). Endorsing obama licenses favoring whites. *J. Exp. Soc. Psychol.* 45 590–593. 10.1016/j.jesp.2009.02.001

[B13] Effron D. A. Monin B. (2010). Letting people off the hook: when do good deeds excuse transgressions? *Pers. Soc. Psychol. Bull.* 36 1618–1634. 10.1177/0146167210385922 20978222

[B14] Ehrhart M. G. Naumann S. E. (2004). Organizational citizenship behavior in work groups: a group norms approach. *J. Appl. Psychol.* 89 960–974. 10.1037/0021-9010.89.6.960 15584835

[B15] Ethics Resource Center. (2013). *National Business Ethics Survey of the U.S. Workforce 2013.* Available at: http://www.ethics.org/ecihome/research/nbes/nbes-reports/nbes-2013

[B16] Festinger L. (1957). *A Theory of Cognitive Dissonance.* Stanford, CA: Stanford University Press.

[B17] Goldstein N. J. Cialdini R. B. (2007). The spyglass self: a model of vicarious self-perception. *J. Pers. Soc. Psychol.* 92 402–417. 10.1037/0022-3514.92.3.402 17352600

[B18] Hollander E. P. (1958). Conformity, status, and idiosyncrasy credit. *Psychol. Rev.* 65 117–127. 10.1037/h0042501 13542706

[B19] Howell J. M. Avolio B. J. (1992). The ethics of charismatic leadership: submission or liberation? *Executive* 6 43–54.

[B20] Johnson D. E. Erez A. Kiker D. S. Motowidlo S. J. (2002). Liking and attributions of motives as mediators of the relationships between individuals’ reputations, helpful behaviors and raters’ reward decisions. *J. Appl. Psychol.* 87 808–815. 10.1037/0021-9010.87.4.808 12184583

[B21] Johnson R. E. Venus M. Lanaj K. Mao C. Chang C. H. (2012). Leader identity as an antecedent of the frequency and consistency of transformational, consideration, and abusive leadership behaviors. *J. Appl. Psychol.* 97 1262–1272. 10.1037/a0029043 22730903

[B22] Joosten A. Van Dijke M. Van Hiel A. De Cremer D. (2013). Feel good, do-good!? On consistency and compensation in moral self-regulation. *J. Bus. Ethics* 123 71–84. 10.1007/s10551-013-1794-z

[B23] Jordan J. Mullen E. Murnighan J. K. (2011). Striving for the moral self: the effects of recalling past moral actions on future moral behavior. *Pers. Soc. Psychol. Bull.* 37 701–713. 10.1177/0146167211400208 21402752

[B24] Kalshoven K. Boon C. T. (2012). Ethical leadership, employee well-being, and helping. *J. Pers. Psychol.* 11 60–68. 10.1027/1866-5888/a000056

[B25] Khan U. Dhar R. (2006). Licensing effect in consumer choice. *J. Mark. Res.* 43 259–266. 10.1509/jmkr.43.2.259

[B26] Kim W. G. Brymer R. A. (2011). The effects of ethical leadership on manager job satisfaction, commitment, behavioral outcomes, and firm performance. *Int. J. Hosp. Manag.* 30 1020–1026. 10.1016/j.ijhm.2011.03.008

[B27] Klotz A. C. Bolino M. C. (2012). Citizenship and counterproductive work behavior: a moral licensing view. *Acad. Manag. Rev.* 38 292–306. 10.5465/amr.2011.0109

[B28] Kouchaki M. (2011). Vicarious moral licensing: the influence of others’ past moral actions on moral behavior. *J. Pers. Soc. Psychol.* 101 702–715. 10.1037/a0024552 21744973

[B29] Krumm A. J. Corning A. F. (2008). Who believes us when we try to conceal our prejudices? The effectiveness of moral credentials with in-groups versus out-groups. *J. Soc. Psychol.* 148 689–710. 10.3200/SOCP.148.6.689-710 19058658

[B30] Lin S. H. J. Ma J. Johnson R. E. (2016). When ethical leader behavior breaks bad: how ethical leader behavior can turn abusive via ego depletion and moral licensing. *J. Appl. Psychol.* 101 815–830. 10.1037/apl0000098 26867103

[B31] Liu D. Liao H. Loi R. (2012). The dark side of leadership: a three-level investigation of the cascading effect of abusive supervision on employee creativity. *Acad. Manag. J.* 55 1187–1212. 10.5465/amj.2010.0400

[B32] Lord R. G. Phillips J. S. Rush M. C. (1980). Effects of sex and personality on perceptions of emergent leadership, influence, and social power. *J. Appl. Psychol.* 65 176–182. 10.1037/0021-9010.65.2.176

[B33] Lowry P. B. Moody G. D. Galletta D. F. Vance A. (2013). The drivers in the use of online whistle-blowing reporting systems. *J. Manag. Inform. Syst.* 30 153–190. 10.2753/MIS0742-1222300105

[B34] Martinko M. J. Harvey P. Sikora D. Douglas S. C. (2011). Perceptions of abusive supervision: the role of subordinates’ attribution styles. *Leadersh. Q.* 22 751–764. 10.1016/j.leaqua.2011.05.013

[B35] Miller D. T. Effron D. A. (2010). Chapter three-psychological license: when it is needed and how it functions. *Adv. Exp. Soc. Psychol.* 43 115–155.

[B36] Monin B. Miller D. T. (2001). Moral credentials and the expression of prejudice. *J. Pers. Soc. Psychol.* 81 33–43. 10.1037/0022-3514.81.1.33 11474723

[B37] Neubert M. J. Carlson D. S. Kacmar K. M. Roberts J. A. Chonko L. B. (2009). The virtuous influence of ethical leadership behavior: evidence from the field. *J. Bus. Ethics* 90 157–170. 10.1007/s10551-009-0037-9

[B38] Nisan M. (1991). “The moral balance model: theory and research extending our understanding of moral choice and deviation,” in *Handbook of Moral Behavior and Development (Volume 3): Application*, eds Kurtines W. M. Gewirtz J. L. (Hillsdale, NJ: Erlbaum), 213–249.

[B39] Ormiston M. E. Wong E. M. (2013). License to ill: the effects of corporate social responsibility and CEO moral identity on corporate social irresponsibility. *Pers. Psychol.* 66 861–893. 10.1111/peps.12029

[B40] Pastoriza D. Ariño M. A. Ricart J. E. (2008). Ethical managerial behaviour as an antecedent of organizational social capital. *J. Bus. Ethics* 78 329–341. 10.1007/s10551-006-9334-8

[B41] Podsakoff P. M. MacKenzie S. B. Lee J. Y. Podsakoff N. P. (2003). Common method biases in behavioral research: a critical review of the literature and recommended remedies. *J. Appl. Psychol.* 88 879–903. 10.1037/0021-9010.88.5.879 14516251

[B42] Riggio R. E. Zhu W. Reina C. Maroosis J. A. (2010). Virtue-based measurement of ethical leadership: the leadership virtues questionnaire. *Consult. Psychol. J. Pract. Res.* 62 235–250. 10.1037/a0022286

[B43] Sachdeva S. Iliev R. Medin D. L. (2009). Sinning saints and saintly sinners: the paradox of moral self-regulation. *Psychol. Sci.* 20 523–528. 10.1111/j.1467-9280.2009.02326.x 19320857

[B44] Shamir B. Kark R. (2004). A single-item graphic scale for the measurement of organizational identification. *J. Occup. Organ. Psychol.* 77 115–123. 10.1348/096317904322915946

[B45] Shapiro D. L. Boss A. D. Salas S. Tangirala S. Von Glinow M. A. (2011). When are transgressing leaders punitively judged? An empirical test. *J. Appl. Psychol.* 96 412–422. 10.1037/a0021442 21133529

[B46] Tajfel H. Turner J. C. (1979). “An integrative theory of intergroup conflict,” in *The Social Psychology of Intergroup Relations*, eds Austin W. G. Worchel S. (Monterey, CA: Brooks), 33–48.

[B47] Tangney J. P. Stuewig J. Mashek D. J. (2007). Moral emotions and moral behavior. *Ann. Rev. Psychol.* 58 345–372. 10.1146/annurev.psych.56.091103.070145 16953797 PMC3083636

[B48] Tepper B. J. (2000). Consequences of abusive supervision. *Acad. Manag. J.* 43 178–190.

[B49] Tepper B. J. (2007). Abusive supervision in work organizations: review, synthesis, and research agenda. *J. Manag.* 33 261–289. 10.1177/0149206307300812

[B50] Wang R. Jiang J. (2015). How abusive supervisors influence employees’ voice and silence: the effects of interactional justice and organizational attribution. *J. Soc. Psychol.* 155 204–220. 10.1080/00224545.2014.990410 25492100

[B51] Wanous J. P. Reichers A. E. Hudy M. J. (1997). Overall job satisfaction: how good are single-item measures? *J. Appl. Psychol.* 82 247–252. 10.1037/0021-9010.82.2.247 9109282

[B52] Whetstone J. T. (2001). How virtue fits within business ethics. *J. Bus. Ethics* 33 101–114. 10.1023/A:1017554318867

[B53] Wilcox K. Vallen B. Block L. Fitzsimons G. J. (2009). Vicarious goal fulfillment: when the mere presence of a healthy option leads to an ironically indulgent decision. *J. Consum. Res.* 36 380–393. 10.1086/599219

[B54] Yam K. C. Klotz A. C. He W. Reynolds S. J. (2017). From good soldiers to psychologically entitled: examining when and why citizenship behavior leads to deviance. *Acad. Manag. J.* 60 373–396. 10.5465/amj.2014.0234

[B55] Zhang Y. Waldman D. A. Han Y. L. Li X. B. (2015). Paradoxical leader behaviors in people management: antecedents and consequences. *Acad. Manag. J.* 58 538–566. 10.5465/amj.2012.0995

[B56] Zheng Q. Wang M. Li Z. (2011). Rethinking ethical leadership, social capital and customer relationship. *J. Manag. Develop.* 30 663–674. 10.1108/02621711111150182

[B57] Zhong C. B. Liljenquist K. Cain D. M. (2009). “Moral self-regulation: licensing and compensation,” in *Psychological Perspectives on Ethical Behavior and Decision Making*, ed. Cremer D. D. (Charlotte, NC: Information Age), 75–89.

